# The Successful Transplanting of a Piece of Rabbit's Cornea into the Human Eye, for the Purpose of Restoring Sight to a Blind Man

**Published:** 1888-07

**Authors:** Julian J. Chisolm

**Affiliations:** University of Maryland.


ARTICLE III.

### THE SUCCESSFUL TRANSPLANTING OF A PIECE OF RABBIT'S CORNEA INTO THE HUMAN EYE, FOR THE PURPOSE OF RESTORING SIGHT TO A BLIND MAN.

BY PROF. JULIAN J. CHISOLM, M. D., UNIVERSITY OF MARYLAND.

Robert Gross, aged 29, a short healthy man, lost both of his eyes three years since from the effects of caustic lime. In each eye the conjunctiva sloughed from over the entire face of the cornea and parts of the lids, leaving the upper lid adherent to the whole corneal surface in the left eye, and both lids firmly adherent to each other and to the eye-ball in the right eye. One year since I liberated the upper lid in the left eye by facing it with a large flap of fleshly substance dissected up from the cornea. Some weeks afterwards I liberated the right lid, first from the lower one, and then from the surface of the cornea. Finding no mucous membrane to face the lid with I prevented the reunion of lid and cornea by using a long tongue of skin from the outer surface of the whole length of the upper lid. After dissecting up this skin flap I made at its root near the outer canthus a vertical slit through the whole thickness of the lid. I then drew the long tongue of skin through the slit, and twisting the pedicle on itself so as to make its raw surface come in contact with the raw surface of the lid secured it in position by sutures. This novel substitution succeeded well. It now permits this eye to open and expose its cornea.

These operations did not, however, improve the transparency of the cornea. Granulations sprang up upon the raw surface, and soon each cornea was again covered entirely by a fleshly conjunctiva, shutting out all chances for useful vision. There was every reason to believe that all other parts of the eye, except the opaque cornea, were normal. There was good light perception but no defination whatever. The case was deemed incurable after a second attempt had been made on each eye to remove the obsuring surface from the cornea by dissecting off this fleshy layer.

Recently Dr. L. Webster Fox, of Philadelphia, had performed the operation first suggested and executed by Prof. von Hippel, of Giessen, Germany, of transplanting a piece of healthy rabbit's cornea into a hole cut out to receive it in the opaque cornea of the human subject. The rabbit is selected, because it is an animal readily obtained, is easily manipulated, and has a large eye. The cornea of any animal could be equally used. My patient seemed one peculiarly adapted to this experiment. Dr. Fox explained to me the mode of operation and very kindly offered me the use of the special apparatus of Prof. von Hippel for making the section, without which the operation could not be successfully performed.

The operation is a speces of skin grafting. Skin grafting I had often done successfully, both in making eye lids, and in adding to the conjunctival surface to prevent the readhesion of the lid in symblepharon. Attempts had been made at corneal transplantation but with no success, because the transplanted piece, however carefully adjusted, always sloughed away, or would be washed away by the ever escaping aqueous secretion. To make a successful graft the anterior chamber must be kept intact. Up to the time of von Hippel's operations this was a factor impossible to be obtained. The step of all importance in preparing the place for the graft is to remove a circular piece of corneal tissue down to Descemet's membrane, leaving this delicate layer of the lining membrane of the anterior chamber undisturbed. The eye ball must remain full, with no leakage whatever of aqueous to disturb the growing together of the graft and its bed. Fortunately the anatomy of the eye readily admits of this. The various layers of the cornea are much more intimately united to each other than is the innermost layer to the membrane of Descemet. So that if these layers all be divided the plug of cornea can be readily separated from the basement membrane or lining membrane of the aqueous chamber. The trephine worked by wrist movement can not affect this. Prof. von Hippel's ingenious trephine permits this essential part of the operation to be perfected.

The instrument itself is a modification of the well-known artificial leech. The circular knife is made to revolve by clock work, placed in the handle of the instrument, and yet not giving much weight to it. The slightest pressure upon a small knob in the head of the handle starts the clock movement and causes a rapid revolution of the circular knife. With this instrument not only is the piece cut out from the human cornea, but a duplicate piece of identical size is cut out of the clear cornea of the rabbit, and the patch must therefore accurately fit into the bed, prepared to receive it. It only needed dexterity in the use of the instrument to make a successful experiment.

Familiar as I am in the use of delicate eye instruments, I anticipated no trouble in going through the various steps of operation. I wisely concluded, however, to begin on the eye of a sheep. From the slaughter house I obtained a couple of fresh heads thinking that operating on these four eyes would give me all the needful experience. It did not require many minutes to show me that I could destroy eyes with the greatest expedition. To hold the delicate trephine so vertically as to cut layer by layer of the cornea and not cut at once, by irregular one-sided pressure, through into the anterior chamber, could only be required after much practice. Many hours and a great many eyes were made use of before this delicacy of manipulation was required. Finally I found myself able to remove the circle of corneal tissue satisfactorily and leave the membrane of Descemet intact. When I had acquired this facility I

could also go through this membrane uniformly and take out an entire plug of cornea, which was an important part of the operation in the preparation of the graft.

All being now in readiness, on June, the 5th, the delicate operation of transplanting a clear piece of rabbit's cornea into the opaque cornea of the blind patient was undertaken at the Presbyterian Eye and Ear Charity Hospital of Baltimore. When the patient was placed upon the operating table and the lid space filled with cocaine, a large rabbit was well secured to a vivisection frame and his eye also filled with the cocaine solution. Just before the section the conjunctival cavity of each eye was filled with the mercurical antiseptic solution in constant use at the Hospital. The trephine knife and other instruments had been put in absolute alcohol so as to guard as far as possible against inflammatory sequelæ. The trephine was applied vertically to the centre of the cornea and a superficial groove three sixteenths of an inch in diameter was made. As the surface was very vascular, blood oozed freely at first, necessitating constant sponging to enable me to reapply the instrument in the groove so as to cut layer by layer of corneal tissue. Upon inspection it was readily seen that where the cornea had been cut sufficiently deep the edges of the circular piece would shrink away from its opposing wall and cup inwards. In the reapplication of the trephine a little more pressure would then be made in the opposite direction for the purpose of avoiding injury to the membrane of Descemet. Finally the entire circular flap seemed loose, by its general shrinkage from the cut edge of the cornea. One edge of it was seized with an iris forceps and the circular flap was peeled out of its position, the adhezion between the lower layers of the corneal plug and Descemet's membrane yielding readily to the traction. The clean uninjured membrane of Descemet came beautifully into view and through it the patient saw for the first time in three years.

The receptacle being now ready, attention was turned to the rabbit's eye. The graft had to be of the entire thickness of the cornea including the membrane of Descemet. In a few seconds this was completely cut through, the trephine sinking deeply into the acqueous chamber. When the instrument was removed the graft did not come away in the hollow of the trephine as was expected, but was left free completely separated, in the opening through the cornea, and was taken out by a delicate forceps. Its Descemet surface was readily recognized by its concavity, the flap showing distinctly its concavo-convex surfaces.

The adjustment in the hole of the human cornea was soon made and the accuracy of the fit was very satisfactory.

The rabbit's cornea is thinner than the human. The two membranes of Descemet gave the proper thickness and established the continuity of the anterior surface. By suction as it were, the graft stuck in its new position. As there had been no perforation of the anterior chamber there was no escape of aqueous to disturb or dislodge the graft. The lids was closed by compress and bandage, with the usual antiseptic precautions in general use at the Hospital.

For five days the eyes remained undisturbed. The patient had no discomfort whatever, nor evidences of any inflammatory reaction except in itching on the fifth day. When the compress was removed the cloth was found somewhat matted from mucus discharges during the five days of its presence on the eye. The secretions were removed from the lash border by a soft sponge wetted with the antiseptic solution. When the lids were separated the graft was found firmly united throughout ¾ of its circumference. At one side there was a little groove of separation visible. As its lower face was uniformly united to the human Descemet's membrane consolidation was perfect. During the process of union the graft had lost its transparency. Through the opaque patch the patient could discern no object, only a flood of light, very much greater than at any time during his three years of blindness.

As the operation was a novel one, and so far successfully completed, I concluded to duplicate the same on the right eye also. In this case as in the previous operation all the layers of the corneal tissue proper were removed leaving the membrane of Descemet undisturbed. At the end of five days I found my second graft equally united to the centre of the right cornea in the hole prepared for it by trephining. This graft so transparent when inserted had also become cloudy during the process of union to the contiguous human cornea. Both eyes somewhat resemble the placing of a pearl in the centre of the pannitic surfaces.

These successful operations in the same subject establish a very important advance in ophthalmic surgery, viz., that with proper precautions a corneal graft from an animal can be successfully transplanted into the human eye, and that the absence of blood-vessels in the graft is not an obstacle to its fusion with contiguous living tissue. To what extent the vascular condition of the cornea of these pannitic eyes aided in the speedy union of the graft is a matter for consideration. The liberal supply of blood-vessels, making a vascular wall in contact with the edges of the corneal graft, must have aided materially in nourishing it and finally in establishing the direct growing together of these contiguous surfaces. The serous surface of Descemet on the graft united promptly with the exposed upper surface of Descemet's membrane of the human eye. It was an epithelial layer of a serous surface coming in contact with a raw surface and prompt union occurred. Union could not have taken place had the graft been inverted, turning its conjunctival layer inwards.

The next point of great interest is as to the extent of clearing of these grafts. Will they regain their transparency, now that the infiltration so needful during the process of primary union of the animal graft to the human cornea is no longer necessary? This process of clearing up has already started in the eye first operated upon. When the bandages were removed only a flood of light was experienced. Three weeks have now elapsed since the first operation. Light is commencing to stray through the thining flap in sufficient amount to cast shadows on the retina. Already the blind man can detect large objects as they move in several feet front of him. From the progress made in two weeks there is every reason to expect that in a short time he will have vision enough to guide himself, when for three years he has been led about.

Each of these eyes was in every way normal except the thick cornea. From the membrane of Descemet back to the retina all structures are sound. Now a healthy piece of cornea, the full size of the pupil is inserted over the pupilary area. It has become firmly united to the human eye, and is as much a part of it as if the man had been born with it. Already the improvement in the recognition of objects, however dimly, amply repays the patient for the anxieties of the experiment. His daily experience shows a slowly improving sight and holds out a very strong hope that he is going to regain much that he formerly enjoyed. Further changes as they become visible to patient or surgeon will be carefully noted and reported.

It is sincerely hoped that this successful experiment in conservative surgery may give so much benefit to a class of hopelessly blind people as to place corneal transplanting in the list of available operations.—*Md. Med. Journal.*

